# 

**Published:** 1884-02-20

**Authors:** 


### TABLE OF CONTENTS.

ORIGINAL AND SELECTED ARTICLES:

The Immediate Removal of the Secundines After Abortion, by Arch Dixon, M.D., Henderson, Ky., 41. Diphtheria, by R. S. Powell, M.D., of Virginia, 48. The More Recent Methods of Treating Wounds, 50. Prognosis in Various Forms of Paralysis, by Philip Zenner, A. M., M.D., 57. The Oxytocic Action of Quinine, 60

ABSTRACTS AND GLEANINGS:

European vs. American Surgeons, 61. Medicine by Innunction, 62. Collodion, 63. The Treatment of Delirium Tremens, 64. Treatment of Fractures by Plaster of Paris Dressing, 65. The True Nature of a Cold, 66. Antiseptics in the Puerpera: On the Action of Agaricin in the Night sweats of Phthisis, 67. Treatment of Exophthalmic Goitre; Vesication in Diphtheria; Corrosive Sublimate in Gonorrhœa, 68. A New Treatment for Neuralgia; Sure Cure for Corns; Yellow Oxide of Mercury in Ophthalmia, 69. Tetanus Cured; Caution about Belladonna Plasters; New Method of Tooth-Drawing, 70.

SCIENTIFIC ITEMS:

Influence of Science on Religion, 71. The Strange Condition; The Approaches; In his Report; Easy writing, 72.

PRACTICAL NOTES AND FORMULÆ.

Suppositories for Piles; Formula for Hostetter's Bitters; Eczema—Acute; For Asthma, 73. Glycerite of Tar; For Neuralgia; Calomel as a Rat Poison, 74. Needless, Useless Coughing; Asthma Mixture; Itch Ointment; Cough Mixture; For Typhoid Fever; How to Give Quinine to Children, 75. A Pleasant Combination of Salicylate of Sodium; Syrup of Coffee; A Liniment for Rheumatism; For Irritable Bladder; Anodyne; Aphrodisiac, 76.

EDITORIALS AND MISCELLANEOUS.

Editorial Notices; Southern Medical College, Atlanta; Medical Association of Georgia, 77. Bacillus of Tubercle; The Physician and the State; Book Notices, 78. Pamphlets and Books Received, 79. Receipted; Special Notices, 80.

